# The Effects of Captivity on the Mammalian Gut Microbiome

**DOI:** 10.1093/icb/icx090

**Published:** 2017-08-07

**Authors:** Valerie J. McKenzie, Se Jin Song, Frédéric Delsuc, Tiffany L. Prest, Angela M. Oliverio, Timothy M. Korpita, Alexandra Alexiev, Katherine R. Amato, Jessica L. Metcalf, Martin Kowalewski, Nico L. Avenant, Andres Link, Anthony Di Fiore, Andaine Seguin-Orlando, Claudia Feh, Ludovic Orlando, Joseph R. Mendelson, Jon Sanders, Rob Knight

**Affiliations:** 1 Department of Ecology and Evolutionary Biology, University of Colorado at Boulder, CO, USA; 2 Department of Pediatrics and Computer Science & Engineering, University of California at San Diego, CA, USA; 3 Institut des Sciences de l’Evolution, Université de Montpellier, UMR 5554, CNRS, IRD, EPHE, France; 4 Department of Anthropology, Northwestern University, IL, USA; 5 Department of Animal Sciences, Colorado State University, CO, USA; 6 National Scientific and Technical Research Council (CONICET), Estacion Biologica Corrientes, Argentina; 7 Department of Mammalogy, National Museum, Bloemfontein, South Africa; 8 Centre for Environmental Management, University of the Free State, Bloemfontein, South Africa; 9 Departamento de Ciencias Biologicas, Universidad de Los Andes, Bogotá, Colombia; 10 Department of Anthropology, University of Texas Austin, TX, USA; 11 Centre for GeoGenetics, Natural History Museum of Denmark, University of Copenhagen, Denmark; 12 National High-Throughput DNA Sequencing Center, University of Copenhagen, Denmark; 13 Association pour le cheval de Przewalski: TAKH, Station Biologique de la Tour du Valat, Arles 13200, France; 14 Zoo Atlanta, GA, USA; 15 School of Biological Sciences, Georgia Institute of Technology, GA, USA; 16 Center for Microbiome Innovation, University of California at San Diego, La Jolla, CA, USA

## Abstract

Recent studies increasingly note the effect of captivity or the built environment on the microbiome of humans and other animals. As symbiotic microbes are essential to many aspects of biology (e.g., digestive and immune functions), it is important to understand how lifestyle differences can impact the microbiome, and, consequently, the health of hosts. Animals living in captivity experience a range of changes that may influence the gut bacteria, such as diet changes, treatments, and reduced contact with other individuals, species and variable environmental substrates that act as sources of bacterial diversity. Thus far, initial results from previous studies point to a pattern of decreased bacterial diversity in captive animals. However, these studies are relatively limited in the scope of species that have been examined. Here we present a dataset that includes paired wild and captive samples from mammalian taxa across six Orders to investigate generalizable patterns of the effects captivity on mammalian gut bacteria. In comparing the wild to the captive condition, our results indicate that alpha diversity of the gut bacteria remains consistent in some mammalian hosts (bovids, giraffes, anteaters, and aardvarks), declines in the captive condition in some hosts (canids, primates, and equids), and increases in the captive condition in one host taxon (rhinoceros). Differences in gut bacterial beta diversity between the captive and wild state were observed for most of the taxa surveyed, except the even-toed ungulates (bovids and giraffes). Additionally, beta diversity variation was also strongly influenced by host taxonomic group, diet type, and gut fermentation physiology. Bacterial taxa that demonstrated larger shifts in relative abundance between the captive and wild states included members of the Firmicutes and Bacteroidetes. Overall, the patterns that we observe will inform a range of disciplines from veterinary practice to captive breeding efforts for biological conservation. Furthermore, bacterial taxa that persist in the captive state provide unique insight into symbiotic relationships with the host.

## Introduction

The mammalian gut microbiome provides a range of essential functions for the host from digestion of complex food to signaling the host immune system (McFall-Ngai etal. 2013). While host phylogeny and diet are both known to shape the composition and function of mammalian gut bacterial communities (Ley etal. 2008; Muegge etal. 2011; Delsuc etal. 2014), changes in living environment also are likely to have a large influence on the microbiome. However, few studies have yet to address this, and those that do focus on relatively few target species (e.g., Kohl and Dearing 2014; Clayton etal. 2016; Kueneman etal. 2016). For most animals, captivity in human-constructed environments (rehabilitation, breeding, pet industry facilities, zoos, etc.) represents an extreme change from the living environment in the wild. In captivity, animals experience many changes that likely impact the microbiome, including changes or restrictions in diet, antibiotic and other veterinary medical interventions, sharply reduced range, reduced contact with a variety of habitat types, reduced interactions with other species, and increased exposure to human-associated microbes and microbes that thrive in a built environment (e.g., Hyde etal. 2016).

Understanding the broad effects of captivity on the microbiome is important for several reasons. First, maintaining animal health in captivity is a top concern for many facilities, and we are only beginning to develop an understanding of what comprises a “healthy microbiome” or range of “healthy microbiomes” for different animals. Second, the few previous studies that directly compared captive to wild counterparts within a species suggest a trend towards reduced symbiotic bacterial diversity in captivity (Loudon etal. 2014; Kohl and Dearing 2014; Clayton etal. 2016; Kueneman etal. 2016), which leads to a number of questions. Is reduced microbiota diversity in captivity a broad trend across animal groups? What is the effect of reduced diversity of the symbiotic microbes in terms of function and host health? Which microbes observed in the wild state persist in captivity, and do the persistent microbes reflect deeper symbiotic ties with the host, in terms of the host's underlying genetically based ability to recruit and retain those microbes (e.g., Van Opstal and Bordenstein 2015)? Thus, by conducting comparative analyses of animal-associated microbiomes in the captive versus wild state, we can begin to address some important knowledge gaps. From a practical standpoint, animal microbiome studies have sometimes used samples from animals in both the captive and wild state (e.g., Ley etal. 2008; Muegge etal. 2011). It is important to gain perspective on the effect captivity has on the microbiome, and incorporate this knowledge into future animal microbiome study design.

For the present study, we targeted mammal species spanning the diversity of the mammalian tree, and obtained paired samples from the captive and wild state for each mammalian taxon, paired at the species, genus, or family level. This effort involved field collections from wild populations ranging from South America to Africa and Mongolia, and collections from a network of accredited zoos in North America and Europe. By sequestering all the samples and processing them using the same standardized protocols, and DNA sequencing instrument, we have reduced as much of the noise that could be attributed to sample processing as possible. Our dataset includes 41 mammal species from several Orders (aardvarks, anteaters, primates, carnivores, and even and odd-toed ungulates), enabling us to examine a range of host traits such as diet type, gut fermentation type, body size, etc. as co-factors of gut bacteria change in captivity. Our dataset provides a coarse level perspective on the effects of captivity on the mammalian gut bacteria and guides future questions. The main questions we address in this study are: (1) Is the loss of gut bacteria diversity in captivity a general pattern? (2) What host traits are associated with either large changes or stability of the gut bacteria in captivity? (3) Do particular bacteria tend to increase or decrease in relative abundance in captivity?

## Methods

### Sample collection

Through a collaborative network, we gathered fecal samples from 41 species of wild and captive mammals (Table 1, Supplementary Table S1). Supplementary Tables S2 and S3 describe how mammalian taxa were grouped for downstream analyses. Mammals sampled span several orders and represent a range of body sizes, diet types, and gut physiologies, allowing for comparison of these host traits in the context of captivity. Samples from captive mammals were collected from eight different zoos: National Zoo (USA), Zoo Atlanta (USA), St Louis Zoo (USA), Beauval Zoo (France), Montpellier Zoo (France), Toulon Zoo (France), Sigean African Reserve (France), and Zurich Zoo (Switzerland) (see Supplementary Table S1). Wild samples were collected by several of the authors (FD, NA, KA, MK, AL, TD, ASO, CF, LO) from wild mammal populations in Central America, South America, South Africa, and Mongolia. For both captive and wild mammal fecal collections, we operated under an approved IACUC protocol through the University of Colorado and appropriate permits were obtained for both sample collection and export. A subset of samples were originally collected for previously published studies (Ley etal. 2008; Muegge etal. 2011; Delsuc etal. 2014; see Supplementary Table S1) and were reprocessed as necessary to ensure that sequencing protocols were consistent across samples (see Supplementary Table S1). For sampling, up to 2g of fresh fecal material was collected per individual using sterile swabs (BD CultureSwab). In most cases, samples were collected within minutes to hours of deposition, remained untreated and were frozen within a few hours of collection (a few exceptions are noted in Supplementary Table S1), and remained frozen (−20˚C) until DNA extraction.

**Table 1:** List of 41 mammal species and sample numbers from the wild and captive states, organized taxonomically

Host taxonomy	Common name	Captive (*n*)	Wild (*n*)	Total (*n*)	Fermentation type	Diet type
Carnivora						
Canidae						
* Canis lupus*	Wolf	4	0	4	N	C
*Lycaon pictus*	African Wild Dog	1	4	5	N	C
Felidae						
*Acinonyx jubatus*	Cheetah	1	2	3	N	C
Cetartiodactyla						
Bovidae						
*Aepyceros melampus*	Impala	3	3	6	FG	H
*Antidorcas marsupialis*	Springbok	5	4	9	FG	H
*Connochaetes gnou*	Black Wildebeest	1	0	1	FG	H
*Connochaetes taurinus*	Blue Wildebeest	5	2	7	FG	H
*Hippotragus equinus*	Roan Antelope	1	0	1	FG	H
*Hippotragus niger*	Sable Antelope	4	2	6	FG	H
Giraffidae						
*Giraffa camelopardalis*	Giraffe	4	2	6	FG	H
Suidae						
*Phacochoerus africanus*	Common Warthog	1	4	5	HG	O
Perissodactyla						
Equidae						
*Equus asinus*	African Wild Ass	5	0	5	HG	H
*Equus quagga*	Plains Zebra	4	2	6	HG	H
*Equus grevyi*	Greyvi’s Zebra	3	0	3	HG	H
*Equus hemionus*	Onager	3	0	3	HG	H
*Equus przewalskii*	Przewalski’s Horse	4	4	8	HG	H
*Equus zebra*	Mountain Zebra	3	3	6	HG	H
Rhinocerotidae						
*Ceratotherium simum*	White Rhinoceros	3	3	6	HG	H
*Diceros bicornis*	Black Rhinoceros	6	1	7	HG	H
Pilosa						
Myrmecophagidae						
*Myrmecophaga tridactyla*	Giant Anteater	11	30	41	N	C
Primates						
Atelidae						
*Alouatta caraya*	Black Howler	0	12	12	HG	O
*Alouatta palliata*	Mantled Howler	0	12	12	HG	O
*Alouatta pigra*	Guatemalan Black Howler	2	13	15	HG	O
*Alouatta seniculus*	Venezuelan Red Howler	0	10	10	HG	O
*Ateles belzebuth*	White-bellied Spider Monkey	0	5	5	N	O
*Ateles fusciceps*	Black-headed Spider Monkey	2	0	2	N	O
*Ateles hybridus*	Brown Spider Monkey	0	3	3	N	O
Cercopithecidae						
*Cercopithecus ascanius*	Red-tailed Monkey	1	8	9	N	O
*Cercopithecus cephus*	Moustached Guenon	2	0	2	N	O
*Cercopithecus neglectus*	De Brazza’s Monkey	1	0	1	N	O
*Cercopithecus wolfi*	Wolf’s Guenon	1	0	1	N	O
*Colobus angolensis*	Black and White Colobus	2	0	2	FG	O
*Colobus guereza*	Mantled Guereza	1	8	9	FG	O
*Papio Anubis*	Olive Baboon	0	7	7	N	O
*Papio hamadryas*	Hamadryas Baboon	0	8	8	N	O
*Papio ursinus*	Chacma Baboon	0	2	2	N	O
Hominidae						
*Gorilla gorilla*	Western Gorilla	8	11	19	HG	H
Lemuridae						
*Eulemur rubriventer*	Red-bellied Lemur	0	12	12	N	H
*Eulemur rufus*	Red Lemur	2	0	2	N	H
*Lemur catta*	Ring-tailed Lemur	3	10	13	N	H
Tubulidentata						
Orycteropodidae						
*Orycteropus afer*	Aardvark	18	5	23	N	C

Subsets of these data were used for bacterial alpha and beta diversity analyses, respectively, according to appropriate sample sizes for statistical comparisons (see Supplementary Tables S2 and S3 for specific sample lists used in those analyses). Diet type (C = carnivore, H = herbivore, O = omnivore) are indicated as well as gut fermentation type (FG = foregut fermenter, HG = hindgut fermenter, N = neither fermentation type).

### Sample processing, sequencing, and bioinformatics

DNA extraction and amplification were performed following the protocol outlined by the Earth Microbiome Project (http://www.earthmicrobiome.org/protocols-and-standards/). Briefly, DNA was extracted using a 96-well MoBio PowerSoil DNA extraction kit. DNA amplification of the V4 region of the 16S rRNA gene was performed using the barcoded primer set 515f/806r in triplicate (Caporaso etal. 2012). Amplicons were pooled, cleaned using the MoBio UltraClean PCR Clean-Up Kit, and sequenced on an Illumina HiSeq 2500 sequencing platform in rapid run mode at the University of California San Diego’s Institute for Genomic Medicine (La Jolla, CA USA). Additionally, a subset of samples was sequenced at the University of Colorado Biofrontiers Institute’s Next-Generation Genomics Facility (Boulder, CO USA). Details describing sequencing platforms, locations, and run dates for all samples are noted in Supplementary Table S1.

Sequence data were demultiplexed and quality-filtered using default parameters in QIIME 1.9.1 (split_libraries_fastq.py) (Caporaso etal. 2010), with an amended quality score cutoff of 19. Sequences were trimmed to 100nt and sub-operational taxonomic units (sOTU) were identified using the Deblur method (Amir etal. 2017). Briefly, the Deblur method estimates exact sequences using an error profile to correct the Illumina platform sequencing error rate of ∼0.1% per nucleotide, which can cause a proliferation of spurious OTUs and inaccurate taxonomic assignments. sOTUs of low abundance (sum to <25 reads total) were removed, and taxonomy was assigned using the RDP classifier and the Greengenes August 2013 release as the reference database.

Prior to downstream analysis, sOTUs identified as chloroplast and mitochondrial were removed, resulting in a range of 9101–155,415 sequences per sample. Samples were rarefied to 9100 sequences per sample. Statistical analyses were performed in R (R Core Team 2016) using several packages including mctoolsr (https://github.com/leffj/mctoolsr/), vegan (Oksanen etal. 2016), and ggplot2 (Wickham 2009).

### Alpha diversity analyses

To examine patterns of alpha diversity in captive versus wild mammals, we computed alpha-diversity using the Shannon diversity index for each individual sampled, using QIIME 1.9.1. Host mammals were grouped as captive or wild at the level of family (see Supplementary Table S2). Hosts were excluded from analysis if either captive or wild groupings consisted of fewer than two different host individuals at specified taxonomic levels. Statistics of pairwise captive versus wild counterparts were computed using R’s vegan package (Oksanen etal. 2016) using two-tailed t-tests. Box-and-whisker-plots were also created in R with package ggplot2 v. 2.2.0.

### Beta diversity analyses, categorical, and continuous variables

To examine how host traits are associated with changes in the gut bacteria in captivity, we analyzed beta-diversity patterns using the Bray Curtis dissimilarity distance metric. We considered several host traits including categorical variables (host taxonomy at the genus level, gut fermentation type, diet type, conservation status) as well as continuous variables (body mass and diet breadth). Supplementary Table S1 indicates how different mammal species were coded for each trait. Mammal trait information was gathered primarily from two sources including the IUCN redlist (IUCN 2016) and a published database of Elton traits for mammals (Wilman etal. 2014). Diet breadth was calculated by applying Shannon’s diversity index to the Eltonian trait diet categories (fruit, invertebrate, nectar, plant-other, seeds, scavengings, warm blooded vertebrates, fish, and unknown vertebrates; Wilman etal. 2014) as types and the proportion of the diet they made up as their abundances. For the categorical host trait variables of interest we utilized Permanova tests to compare microbial communities between captive and wild groups separately for each host trait variable (Oksanen, 2016, R package version 3.2.2, ADONIS). For a listing of which species were included in beta diversity comparisons, see Supplementary Table S3. Low sample numbers per category prevented us from running multi-factor host trait analyses in most cases. Visualizations of the ordinations were produced using *mctoolsr* package. For the continuous host trait variables (body mass and diet breadth) we used Mantel tests. For each continuous variable we calculated euclidean distance matrices (using “dist” in the base R stats package) and then ran a Mantel test (using the vegan package for R) comparing our microbial community and host trait variable distance matrices. These analyses were performed on both the dataset overall and the captive and wild data separately.

### Bacterial taxa differences in captive versus wild

We determined if any bacterial taxa (OTUs, genera, classes, and phyla) were significantly more abundant in either the captive or wild condition both across all mammals and within mammal genera using Mann–Whitney tests, with an FDR correction. We only included bacterial taxa that were at least 1% abundant on average across samples. Bacteria with no taxonomic assignment at the level being tested were removed from analyses, with the exception of unidentified OTUs (unique OTUs without an assigned taxonomy), which were included. Analyses were performed using R program v. 3.3.1 with package *mctoolsr*. Analyses performed per mammalian genus included only those genera with at least two individuals sampled in both the captive and wild state.

## Results

**Fig. 1:**
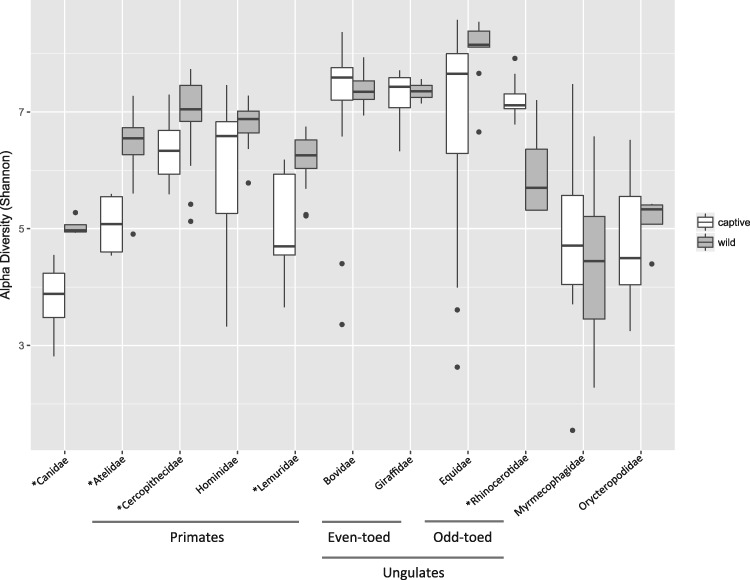
Gut bacterial alpha-diversity comparison between captive and wild mammals. Alpha-diversity was computed in QIIME using the Shannon diversity index per mammal family in the captive and wild state, respectively. See Table 1 for mammal species included in each family. Open bars represent alpha-diversity of microbes within captive hosts; shaded bars represent microbial alpha-diversity within wild hosts. Boxes represent 25–75% quantile with median (50% quantile) represented by a black line; points outside boxes indicate outliers. Two-tailed *t*-tests were used to compare captive versus wild for each mammal family. Asterisks denote significance: *Canidae (*P* = 0.0093), *Atelidae (*P* < 0.001), *Cercopithecidae (*P* = 0.011), Hominidae (*P* = 0.101), *Lemuridae (*P* = 0.0003), Bovidae (*P* = 0.55), Giraffidae (*P* = 0.81), Equidae (*P* = 0.061), *Rhinocerotidae (*P* = 0.0028), Myrmecophagidae (*P* = 0.358), Orycteropodidae (*P* = 0.448).

Differences in bacterial richness (alpha diversity) were observed between the captive and wild states; however, the differences were not consistent across mammalian families (Fig. 1). Two-tailed *t*-tests were used to compare bacterial Shannon diversity in the captive versus wild state for each family. Four mammalian families (comprising 21 species) had significantly decreased gut bacterial diversity in the captive state: Canidae (*P* = 0.0093), Atelidae (*P* = 7.79e-08), Cercopithecidae (*P* = 0.011), Lemuridae (*P* = 0.0003). The Equidae had marginally significantly decreased bacterial diversity in the captive state (*P* = 0.061) and the Hominidae (gorillas) trended toward decreased bacterial diversity (*P* = 0.101). One family, Rhinocerotidae had significantly increased bacterial diversity in captivity (*P* = 0.0028). Four mammal families had no significant change in bacterial diversity between the wild and captive state: Bovidae (*P* = 0.55), Giraffidae (*P* = 0.81) Myrmecophagidae (*P* = 0.358), Orycteropodidae (*P* = 0.448).

**Fig. 2:**
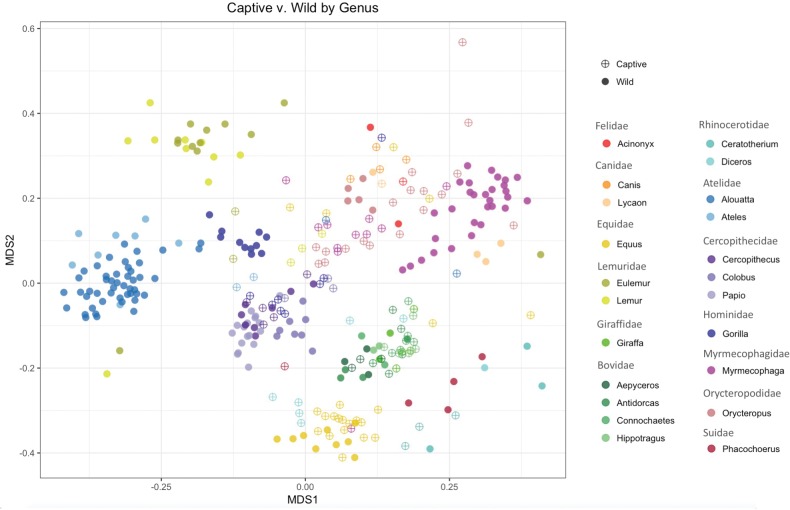
Nonmetric multi-dimensional scaling plot of mammal gut bacterial communities in the captive and wild state, by host genus. Open symbols (with cross-hatch) indicate captive individuals, and closed circles indicate wild individuals. The colors correspond with different mammal genera; similar colors were chosen for host genera of the same family (e.g., shades of navy blue belong to the family Atelidae). Statistical differences in the beta-diversity among captive versus wild per host genus are provided in Table 2.

To examine whether the gut bacterial communities of mammals shift significantly in captivity, we compared changes in beta-diversity in the captive versus wild state. A Permanova (ADONIS) comparing wild versus captive bacterial communities across the whole dataset yielded a significant difference (*P* = 0.001); however, the *R*^2^ value was very low (*R*^2^= 0.024), indicating that the captive/wild factor alone does not explain a large portion of variation in these communities. The categorical variable that explains the highest amount of variation in the dataset is host taxonomy at the genus level (*P* = 0.001, *R*^2^ = 0.405, see Fig. 2). When we examined community differences across the captive versus wild states within each mammal genus, the effect of captivity becomes more apparent (Table 2, Supplementary Table S3). Gut bacterial communities demonstrated significant shifts in the wild versus captive state in 12 out of 15 mammal genera tested; *R*^2^ values ranged from 0.06 to 0.56 with an average *R*^2^ of 0.29 (Table 2). The remaining three genera that did not have significant shifts in beta-diversity between captive and wild groups were all even-toed ungulates (giraffe, impala, and antelope, Table 2).

**Table 2:** Results of Permanova statistics to compare beta-diversity of mammal gut bacterial communities

Host Genus	#perm	Df (factor: total)	SS	MS	F.Model	*R*^2^	*P*
*Eulemur*	999	1:13	1.39	1.39	15.12	0.56	*0.017
*Lemur*	999	1:12	1.49	1.49	11.07	0.50	*0.005
*Gorilla*	999	1:18	2.23	2.23	11.19	0.40	*0.001
*Ateles*	999	1:9	0.89	0.89	4.06	0.34	*0.026
*Antidorcas*	999	1:8	0.65	0.65	3.40	0.33	*0.010
*Colobus*	999	1:10	1.00	1.00	4.29	0.32	*0.006
*Cercopithecus*	999	1:12	0.76	0.76	3.72	0.25	*0.001
*Connochaetes*	999	1:7	0.31	0.31	1.53	0.20	*0.042
*Orycteropus*	999	1:22	1.66	1.66	4.84	0.19	*0.001
*Ceratotherium_Diceros*	999	1:12	0.82	0.82	2.51	0.19	*0.017
*Myrmecophaga*	999	1:40	1.95	1.95	5.57	0.12	*0.001
*Equus*	999	1:30	0.60	0.60	1.80	0.06	*0.011
*Aepyceros*	719	1:5	0.44	0.44	2.80	0.41	0.100
*Giraffa*	719	1:5	0.45	0.45	2.79	0.41	0.067
*Hippotragus*	999	1:6	0.33	0.33	2.13	0.30	0.052
Diet type							
Carnivore	999	1:75	2.11	2.11	5.26	0.07	*0.001
Herbivore	999	1:186	3.96	3.96	9.06	0.05	*0.001
Omnivore	999	1:43	0.70	0.70	1.99	0.05	*0.021
Fermenter type							
Hindgut	999	1:111	4.13	4.13	10.34	0.09	*0.001
Foregut	999	1:46	1.36	1.36	4.52	0.09	*0.001
Neither	999	1:147	2.19	2.18	4.94	0.03	*0.001

Bray–Curtis distances were used for all analyses presented here. Each row represents a Permanova test of community differences between the captive versus wild state for each mammal genus, diet type, or fermenter type, respectively. Rhinoceros genera were combined into one analysis to improve sample numbers (*Ceratotherium* and *Diceros*). Asterisks indicate statistically significant results, *p*<0.05.

**Fig. 3:**
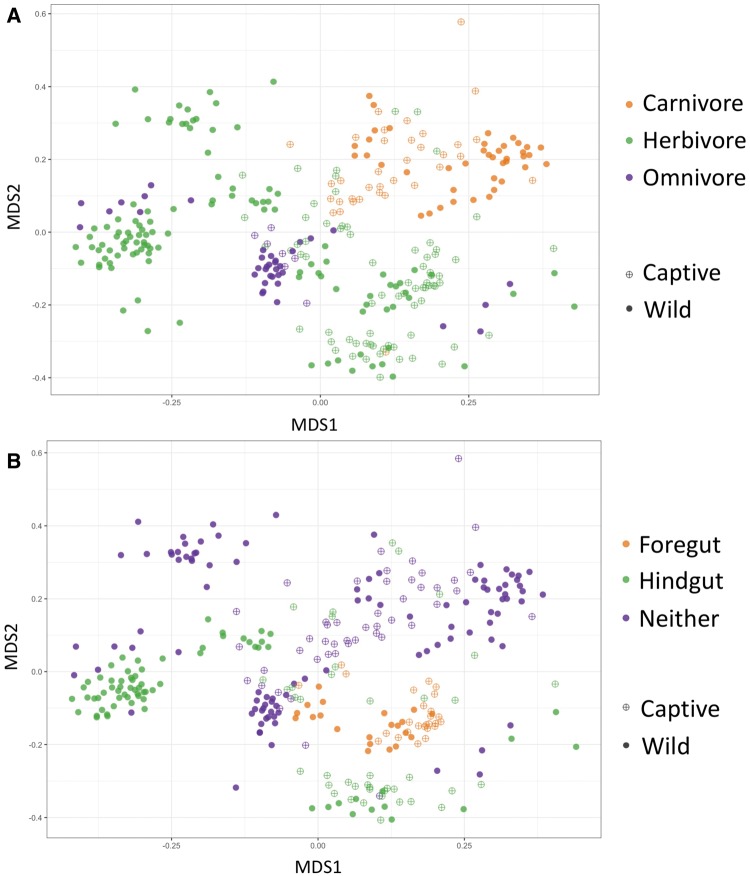
Nonmetric multi-dimensional scaling plot of mammal gut bacterial communities in the captive and wild state, by host diet type (**A**) and host gut fermenter type (**B**). Host trait assignments are listed in Supplementary Table S1. Open circles (with cross hatch) indicate captive individuals, and closed circles indicate wild individuals. In a Permanova analysis, host diet type shown in A, as a sole factor, is a significant predictor of gut bacterial community similarity (*P* < 0.001, *R*^2^ = 0.075), as is host gut fermenter type shown in B (*P* < 0.001, *R*^2^ = 0.091). Beta-diversity differences between the captive versus wild state for these factors are shown in Table 2.

In addition to host taxonomy, host mammal gut fermentation type and diet type also explain significant variability in the gut bacterial communities (Fig. 3, Table S3). Captive versus wild comparisons for each diet and fermenter type also were significantly different; however, the *R*^2^ values are notably lower (Table 2). Conservation status was not a significant effect. We used Mantel tests to examine whether mammal body mass or diet breadth (continuous variables) covaried significantly with the captive/wild state respectively. Results of the Mantel tests indicate that for body mass, there was a positive correlation between body mass differences and bacterial community differences for both captive mammals (*R* = 0.26, *P* = 0.001) and wild mammals (*R* = 0.36, *P* = 0.001). Similarly, we also observed a positive correlation between differences between diet breadth and bacterial community differences for both captive mammals (*R* = 0.15, *P* = 0.001) and wild mammals (*R* = 0.33, *P* = 0.001). All Mantel test correlations performed were positive (greater than zero), and the values of the r statistic were slightly higher for mammals in the wild state versus captive, however, all values were relatively low (≤0.36).

We also examined whether collection site had any large signal in these data, using ADONIS. For example, we were concerned whether captive individuals within a species differed in their microbiome across zoos, creating a potentially confounding effect. Most mammalian genera were sampled from one wild site or from one zoo site, preventing a direct comparison among zoo facilities without being confounded by host taxonomy. However a subset of captive mammals in the dataset (four species and one genus) were sampled from multiple captive sites (see Supplementary Figure S1). Within each mammalian taxon, we ran a 2-way Adonis analysis to examine the effects of collection site (e.g., zoo facility or wild site) and captive/wild. Results indicated that for all five mammals, both collection site and captive/wild were significant factors explaining variation in bacterial gut beta-diversity (Supplementary Table S4). In all cases, the captive/wild factor had a stronger effect size (mean sum of squares) relative to collection site; in the case of gorillas, the effect size for captive/wild was 4.3 times greater than collection site (Supplementary Table S4). Thus, while there are some differences among zoo facilities, because the majority of mammalian genera were sampled from one zoo each, and all wild sample were from one wild site per species, collection site as a factor most likely does not influence the overall trends observed in this large-sale dataset.

**Fig. 4:**
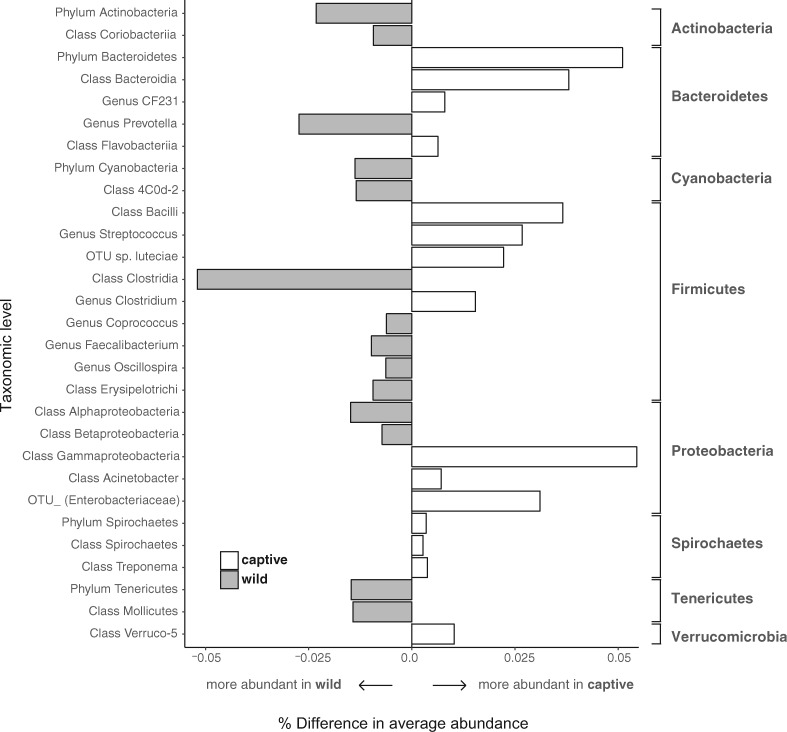
Differences in the average relative abundance of bacterial taxa between captive and wild hosts. Bars represent the percent difference in abundance of mean captive minus mean wild for each bacterial taxa across all mammal host samples. Shaded and open bars indicate significant increases of the relative abundances of specific bacterial taxa in the wild or in captivity, respectively (false discovery rate-corrected *P* < 0.05). Only those bacterial taxa with significant differences at the phyla, class, genus and OTU (97%-cutoff) levels are shown.

**Fig. 5:**
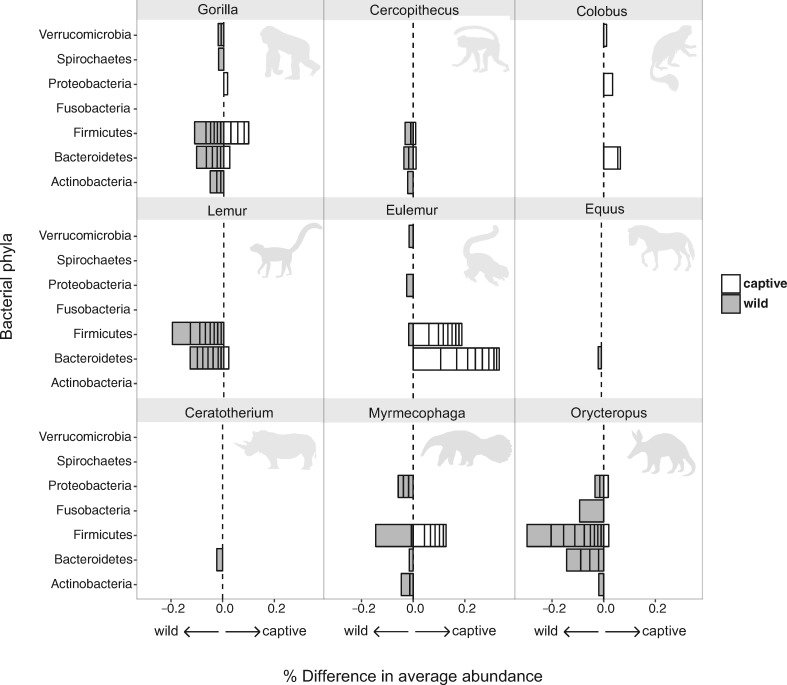
Differences in the relative abundance of bacterial phylotypes between captive and wild hosts, within host genera. Bacterial phylotypes were binned by phyla-level taxonomic identity for each genus plot. Within bins, each segment denotes a bacterial OTU (97%-cutoff) that differed significantly in average relative abundance (false discovery rate-corrected *P* < 0.05). Width of segments shows magnitude of difference in abundance, calculated as captive minus wild. Thus, the overall width of each phyla bin is the cumulative percent difference of significant bacterial OTUs. Shaded and open bars indicate bacterial OTUs with a higher relative abundance in the wild and in captive hosts, respectively. See Supplementary Table S3 for summary statistics and taxonomic identities of bacterial OTUs.

Across the entire mammal dataset encompassing all 41 species, 29 bacterial taxa (at the OTU, genus, class, and phylum levels) were significantly more relatively abundant in either the captive or wild state (Fig. 4). These include members of eight bacterial phyla: Actinobacteria, Bacteroidetes, Cyanobacteria, Firmicutes, Proteobacteria, Spirochaetes, Tenericutes, and Verrucomicrobia. Summary statistics for the taxa that significantly differed in average relative abundance between captive and wild hosts are provided in Supplementary Table S5. At the OTU level, bacterial relative abundance significantly increased or decreased in the captive state for nine mammal genera (Fig. 5). A large proportion of these shifting bacteria belong to the Firmicutes and Bacteroidetes. In some host taxa, these bacteria had a higher relative abundance in the captive state (*Eulemur*), while in others they are more abundant in the wild state (e.g., *Lemur* and aardvarks, Figure 5). Classification of the OTUs that significantly shift in average relative abundance between captive and wild hosts are provided in Supplementary Table S6.

## Discussion

Captivity represents an extreme change in lifestyle for many animal species and given the differences found in human gut microbiomes associated with different lifestyles (Yatsunenko etal. 2012; Schnorr etal. 2014), we would expect to see changes in the microbiome of animals when comparing the wild to the captive state. The small number of studies that have previously addressed this topic examined the gut bacterial communities of a few rodent and primate species (Kohl and Dearing 2014; Clayton etal. 2016) as well as the skin microbiome of a few amphibian species (Loudon etal. 2013; Kueneman etal. 2016). In all of these prior studies, bacterial diversity was significantly reduced in the captive state versus the wild and the authors pointed to reduced diet diversity and reduced contact with variable environmental substrates (e.g., soil and aquatic systems) that may act as sources for diverse bacteria. Our present study encompasses 41 species of mammals across six different Orders that cover the four major placental clades, allowing for a more robust analysis of any generalizable patterns of gut bacterial community changes associated with captivity. Across so many samples from a variety of locations, we were not able to address some factors that may be associated with gut bacterial variation, including animal sex and medical treatments in zoos. We did, however, include samples from mature animals to avoid confounds associated with life stage differences and we processed all the samples using the same standardized protocols, also in an effort to reduce noisy variation. The purpose of our study is to identify coarse level patterns associated with captivity that will stimulate deeper future study. Overall, a clear pattern that emerged was a decrease in bacterial diversity for the primates in this study, wherein four out of five primate families had significantly reduced gut bacterial diversity in captivity, and the remaining family (gorillas) trended toward lower diversity (Fig. 1). Carnivores also showed a pattern of decreased bacterial diversity in captivity, but too few species are represented in this dataset to make a robust conclusion. Equids also demonstrated a pattern of reduced bacterial diversity in captivity that was marginally significant. An important finding of this study, however, is that a decrease in gut bacterial diversity in captivity is not universal across mammals (Figure 1). Several mammalian groups showed no change in bacterial diversity in captivity compared with the wild state (bovids, giraffes, anteaters, and aardvarks). Interestingly, the rhinoceros taxa showed an increase in bacterial gut diversity in captivity. Thus, not all mammals demonstrate the same pattern. We propose that host traits are likely to influence whether a species will experience shifts in the gut bacteria associated with captivity.

Changes in beta-diversity lend further insight into which host traits are associated with either stability or change of the gut bacteria in captivity. Unsurprisingly, host taxonomy strongly predicts gut bacterial community similarity, recapitulating findings from previous comparative studies of mammal gut bacteria (Ley etal. 2008; Muegge etal. 2011; Delsuc etal. 2014). The host signal in the data is strong, such that by examining community change in the captive versus wild state for each mammal genus, we gain better insight into which host taxa display shifts in the gut microbiome in captivity. Here, we observed that most mammal genera in this study have significant changes in their gut bacterial communities associated with captivity (Table 2). Primates exhibited some of the largest changes, as indicated by the larger *R*^2^ values, ranging from 19% to 56% of the variability explained by the captive versus wild state. Several other groups also had significant changes in the gut bacteria in captivity including horses, zebras, wildebeest, springbok, anteaters, aardvarks, and rhinoceros (Table 2). This indicates that some species that did not have decreased bacterial alpha diversity in captivity, such as the anteaters and aardvarks, still differed in terms of bacterial composition and/or relative abundance of various bacterial taxa. Three genera of the even-toed ungulates including impala, giraffe, and antelope did not exhibit gut bacterial community changes in captivity.

### Host traits associated with gut bacteria stability/change in captivity

In terms of host traits that correlate with stability of the gut bacteria in captivity, the even-toed ungulates (Cetartiodactyla) demonstrated the most stability. Several of the cetartiodactylid genera tested did not exhibit changes in bacterial alpha or beta-diversity associated with captivity (Fig. 1, Table 2). These mammals are herbivorous ruminants with foregut fermentation, pre-digesting plant material in the rumen using a rich and dense assortment of anaerobic bacteria. Multiple factors may explain why even-toed ungulates did not show differences in the gut bacterial communities in captivity versus the wild. First, because we sampled feces to characterize the gut bacteria for this study, it is possible that we did not capture differences in the microbiota of the anterior rumen sections of the gut that may be particularly relevant for these ungulates. Rumen sampling is far more invasive and was beyond the scope of this study, and we acknowledge that our sampling scheme may have been insufficient for identifying changes in the gut bacteria that are not adequately represented in fecal samples. Alternatively, if this is not the case, it is possible that even-toed ungulates are suited to captivity in terms of maintaining a wild-like gut bacteria. Indeed, a recent study of ruminant and camelid species from around the world found that a core microbiome exists across ruminants, yet with weak co-association patterns between functional groups (Henderson etal. 2015). The authors suggest that this functional redundancy may mean that the ruminant gut is flexible enough to utilize a variety of feeds. However, it is also likely that this pattern reflects on the institutional knowledge for how to properly balance the diet of these ungulates in captivity. For example, careful attention to the fiber content of the diet is known to help prevent gut dysbiosis in ungulates in captivity (e.g., Clauss and Dierenfeld 2008; Taylor etal. 2013).

A contrasting example to the ungulates is represented by the two myrmecophagous (ant- and termite-eating) species, aardvark and giant anteater, that did not show significant differences in bacterial alpha diversity but did have differences in beta-diversity of the bacterial communities between captive and wild individuals (Fig. 1, Table 2). For anteaters and aardvarks, the diet is markedly different in captivity and zookeepers have had trouble creating a suitable diet for ant-eating mammals to maintain gut health in captivity (Clark etal. 2016). Many captive facilities now use a fully insectivorous diet in the form of a commercial powder called Termant (Mazuri Zoo Foods, Witham, Essex, UK), specifically for giant anteaters and aardvarks. This product was designed to contain essential vitamins and minerals, including chitin and formic acid, that mimic the natural diet requirements. Still, feeding on this artificial diet is far different than feeding on the large quantity of ants and termites that these animals ingest in the wild, which might explain some of the differences in gut bacterial communities observed between our captive and wild individuals (Delsuc etal. 2014).

However, in terms of host traits that correlate with the largest changes of the gut bacteria in captivity, the primates had the largest differences. Carnivores also had significant changes in the gut bacteria associated with captivity, however far fewer carnivore species were represented in this dataset, limiting our ability to confirm a clear pattern. The consistent gut bacteria changes observed in primates are important, as many primate species are highly endangered (Estrada etal. 2017), captive programs are increasingly important, and maintaining primate health in captivity is critical. The finding that primates exhibited the most marked gut bacteria changes associated with captivity is consistent with reports of frequent gastrointestinal illness in captive primates across a range of contexts (Hird etal. 1984; Tucker 1984). Not only might illnesses associated with an altered gut microbiota negatively affect health and reproductive output in captive populations, but survival of individuals reintroduced into the wild from captivity could be negatively affected if the gut microbiota associated with captivity compromises host digestive or immune function in the wild. Interestingly, all primates showed similar microbial responses to captivity despite the wide range of diets and gut morphologies represented. This pattern suggests that more general characteristics shared by all primates are likely responsible for their increased susceptibility to gut microbiota alteration in captivity. Given that all food resources consumed by wild primates tend to be higher in fiber than their domesticated counterparts, reduced fiber intake in captive primates may be an important variable for future exploration. Studies of both rodents and primates indicate that this relatively simple dietary alteration could be responsible for the observed patterns (Clayton etal. 2016; Sonnenburg etal. 2016). Alternatively, reduced contact with complex social networks (e.g., Tung etal. 2015; Amato etal. 2017), as well as increased susceptibility to and treatment for human-associated diseases could impact the gut microbiota of captive primates.

### Bacterial taxa that differ in relative abundance in the wild or captive state

Our dataset allowed us to ask whether specific bacterial groups significantly differ in relative abundance in the captive or wild state across a broad diversity of mammalian taxa. We approached this question at two different scales. First we examined whether bacterial taxa at the levels of phylum, class, genus, and OTU differed across the dataset as a whole, which provides a coarse look at which bacterial taxa tend to shift with captivity in general (Fig. 4, Supplementary Table S5). Next, we scaled down to look at each mammalian genus in the dataset and examine which OTUs shifted significantly between the wild and captive states (Figure 5, Supplementary Table S6). Indeed, many bacterial taxa demonstrated shifts along the wild to captive axis. To summarize generally, shifts in bacteria belonging to the phyla Bacteroidetes, Firmicutes, and Proteobacteria are the dominant players. Below, we discuss a few of the emergent trends from these analyses. The full list of these bacterial taxa, including those not explicitly discussed, can be found in the supplemental material (Supplementary Tables S5–S6).

Within the bacterial phylum Bacteroidetes, a few taxa of interest stand out. Captive mammals in this dataset demonstrated less relative abundance of *Prevotella* overall (although members of the genus *Eulemur* primates were an exception to this pattern). For example, in captive Old World Monkeys (*Cercopithecus*), we observed a significant decrease in relative abundance of both *Prevotella* and *Prevotella copri* and a simultaneous increasing relative abundance of Bacteroidales S24-7. These data support previous hypotheses related to niche space competition between *Prevotella copri* and Bacteroidales S24-7 (Ormerod etal. 2016). *Prevotella* are enriched for in high carbohydrate diets (Wu etal. 2011) and are able to breakdown proteins and carbohydrates (Rosenberg 2014). Starch, xylan, and arabinan are key resources for Bacteroidales S24-7, which are also able to breakdown proteins and carbohydrates (Serino etal. 2012; Evans etal. 2014). In human gut microbiome studies, *Prevotella* are more abundant in children raised on a plant-based rural African diet in comparison with children of similar age/weight sampled within the European Union raised on a higher protein-based diet (De Filippo etal. 2010). *Prevotella* is also significantly reduced in people who shift diets from vegetarian toward solely animal-based foods (David etal. 2014). These trends would indicate that, broadly, a decrease in the relative abundance of *Prevotella* in captive mammals may signal an increase in protein in the captive diet relative to the wild, however, we do not have the diet data to test this idea directly. Another member of the Prevotellaceae family, *Paraprevotella*, decreased in captive rhinoceros as compared to wild (*P* = 0.028). *Paraprevotella* bacteria are obligate anaerobes that are stimulated by xylan to produce succinic and acetic acid as fermentation end products (Morotomi etal. 2009). Though rhinoceros had increased bacterial alpha diversity overall, the reduced abundance of these bacteria in captivity is potentially related to diet changes in captivity.

Within the bacterial phylum Firmicutes, captive mammals demonstrate markedly higher anaerobic Bacilli and lower relative abundance in Clostridia as compared to their wild counterparts. Specifically, our data show a higher relative abundance of *Streptococcus luteciae* and *Clostridium* within captive animals as compared to significantly higher relative abundance of *Coprococcus*, *Faecalibacterium*, and *Oscillospira* in wild individuals. Lactate-producing bacteria, such as *Streptococcus luteciae*, have been associated with overeating of readily fermentable carbohydrates often leading to an imbalanced rumen microbial population and subsequent rumen acidosis (Biddle etal. 2013). In agricultural pig raising facilities, changes in relative abundance of *Prevotella*, CF231, *Ruminococcus*, *Oscillospira*, and *Lactobacillus* have been observed in stool samples from sows that differed only in their housing, specifically with and without straw on pen floors (Kubasova etal. 2017). Thus, in addition to changes in diet associated with captivity, small changes in captive conditions (e.g., floor covering) can significantly influence composition of gut microbiota.

Within the bacterial phylum Proteobacteria, an increase in Gammaproteobacteria (Moraxcellaceae, Enterobacteriaceae) was observed in captive mammals, whereas both Alpha- and Betaproteobacteria were more abundant in wild counterparts. Increased Enterobacteriaceae are observed in high protein, western human diets (De Filippo etal. 2010). This finding, in addition to trends observed within the *Prevotella* taxa, indicates that dietary changes in captivity can have important consequences. It is worth highlighting that wild mammals in this dataset also had significantly increased relative abundance of Cyanobacteria in conjunction with increased Alpha- and Betaproteobacteria, which are known nitrogen reducers. Alpha- and Betaproteobacteria increased in abundance with increased amounts of decaying cyanobacteria within the gut of the buzzer midge (*Chironomus plumosus*; Sun etal. 2014). Thus changes in Proteobacteria may be the result of both direct in and indirect effects related to changes in diet between the wild and captive state

Lastly, captive primates also had an increased relative abundance of Christensenellaceae, a microorganism associated with health (Biagi etal. 2016). Christensenellaceae are known to have high ‘heritability’ (Goodrich etal. 2014) and are thought to be recruited to perform beneficial functions within the gut community (Van Opstal and Bordenstein 2015, reviewed by Fischbach and Segre 2016). We hypothesize that as captive animals lose gut bacteria correlated with health (such as *Prevotella* and Ruminoccoccae), there may be an increased recruitment and proliferation of a highly heritable microbial family (e.g., Christensenellaceae), perhaps as an alternative mechanism to reduce gut dysbiosis and promote gut health; However, it is important to note that, comparisons to findings in the human gut may not translate to other primates and this idea would require further study. Overall, our study provides many leads for important future work.

## Supplementary data

Supplementary data available at *ICB* online.

## Supplementary Material

Supplementary Data
