# Supplementary material for: The Effects of Captivity on the Mammalian Gut Microbiome
Source: Integr Comp Biol. 2017 Aug 7;57(4):690–704. doi: 10.1093/icb/icx090 (PMC5978021; doi:10.1093/icb/icx090)
Supplement: Supplementary Data [file icx090_supp.zip › icx090_SuppTable_2.docx]

Supplementary Table S2. Samples included for comparisons of bacterial alpha diversity across the captive versus wild state were grouped by mammal families (shown in bold). Corresponding analyses are visualized in main text Figure 1. Mammal families that did not include high enough sample sizes were excluded, such as Felidae (cheetahs) and Suidae (warthogs).

| **Host Taxonomy** | **Common Name** | **Captive (n)** | **Wild (n)** | **Total (n)** |
| --- | --- | --- | --- | --- |
| Carnivora |  |  |  |  |
| **Canidae** |  | **5** | **4** | **9** |
| *Canis lupus* | Wolf | 4 | 0 | 4 |
| *Lycaon pictus* | African Wild Dog | 1 | 4 | 5 |
|  |  |  |  |  |
| Cetartiodactyla |  |  |  |  |
| **Bovidae** |  | **19** | **11** | **30** |
| *Aepyceros melampus* | Impala | 3 | 3 | 6 |
| *Antidorcas marsupialis* | Springbok | 5 | 4 | 9 |
| *Connochaetes gnou* | Black Wildebeest | 1 | 0 | 1 |
| *Connochaetes taurinus* | Blue Wildebeest | 5 | 2 | 7 |
| *Hippotragus equinus* | Roan Antelope | 1 | 0 | 1 |
| *Hippotragus niger* | Sable Antelope | 4 | 2 | 6 |
| **Giraffidae** |  | **4** | **2** | **6** |
| *Giraffa camelopardalis* | Giraffe | 4 | 2 | 6 |
|  |  |  |  |  |
| Perissodactyla |  |  |  |  |
| **Equidae** |  | **22** | **9** | **31** |
| *Equus asinus* | African Wild Ass | 5 | 0 | 5 |
| *Equus quagga* | Plains Zebra | 4 | 2 | 6 |
| *Equus grevyi* | Greyvi’s Zebra | 3 | 0 | 3 |
| *Equus hemionus* | Onager | 3 | 0 | 3 |
| *Equus przewalskii* | Przewalski’s Horse | 4 | 4 | 8 |
| *Equus zebra* | Mountain Zebra | 3 | 3 | 6 |
| **Rhinocerotidae** |  | **9** | **4** | **13** |
| *Ceratotherium simum* | White Rhinoceros | 3 | 3 | 6 |
| *Diceros bicornis* | Black Rhinoceros | 6 | 1 | 7 |
|  |  |  |  |  |
| Pilosa |  |  |  |  |
| **Myrmecophagidae** |  | **11** | **30** | **41** |
| Myrmecophaga tridactyla | Giant Anteater | 11 | 30 | 41 |
|  |  |  |  |  |
| Primates |  |  |  |  |
| **Atelidae** |  | **4** | **55** | **59** |
| Alouatta caraya | Black Howler | 0 | 12 | 12 |
| Alouatta palliata | Mantled Howler | 0 | 12 | 12 |
| Alouatta pigra | Guatemalan Black Howler | 2 | 13 | 15 |
| Alouatta seniculus | Venezuelan Red Howler | 0 | 10 | 10 |
| Ateles belzebuth | White-bellied Spider Monkey | 0 | 5 | 5 |
| Ateles fusciceps | Black-headed Spider Monkey | 2 | 0 | 2 |
| Ateles hybridus | Brown Spider Monkey | 0 | 3 | 3 |
| **Cercopithecidae** |  | **8** | **33** | **41** |
| Cercopithecus ascanius | Red-tailed Monkey | 1 | 8 | 9 |
| Cercopithecus cephus | Moustached Guenon | 2 | 0 | 2 |
| Cercopithecus neglectus | De Brazza’s Monkey | 1 | 0 | 1 |
| Cercopithecus wolfi | Wolf’s Guenon | 1 | 0 | 1 |
| Colobus angolensis | Black and White Colobus | 2 | 0 | 2 |
| Colobus guereza | Mantled Guezera | 1 | 8 | 9 |
| Papio anubis | Olive Baboon | 0 | 7 | 7 |
| Papio hamadryas | Hamadryas Baboon | 0 | 8 | 8 |
| Papio ursinus | Chacma Baboon | 0 | 2 | 2 |
| **Hominidae** |  | **8** | **11** | **19** |
| Gorilla gorilla | Western Gorilla | 8 | 11 | 19 |
| **Lemuridae** |  | **5** | **22** | **27** |
| Eulemur rubriventer | Red-bellied Lemur | 0 | 12 | 12 |
| Eulemur rufus | Red Lemur | 2 | 0 | 2 |
| Lemur catta | Ring-tailed Lemur | 3 | 10 | 13 |
|  |  |  |  |  |
| Tubulidentata |  |  |  |  |
| **Orycteropodidae** |  | **18** | **5** | **23** |
| Orycteropus afer | Aardvark | 18 | 5 | 23 |
