# Supplementary material for: The Effects of Captivity on the Mammalian Gut Microbiome
Source: Integr Comp Biol. 2017 Aug 7;57(4):690–704. doi: 10.1093/icb/icx090 (PMC5978021; doi:10.1093/icb/icx090)
Supplement: Supplementary Data [file icx090_supp.zip › icx090_SuppTable_3.docx]

Supplementary Table S3. Samples included for comparisons of bacterial beta diversity across the captive versus wild state were grouped by either mammal genus, diet type, or gut fermenter type, respectively (see bolded rows). Corresponding analyses are visualized in Figures 2 and 3, and statistics are presented in Table 2. Mammal genera that did not include high enough sample sizes were excluded for comparisons at the genus level (e.g, wolves, wild dogs, cheetahs, warthogs, howler monkeys, baboons); However, these mammals were included in groupings by diet type and gut fermenter type.

| **Host Genus / Species** | **Common Name** | **Captive (n)** | **Wild (n)** | **Total (n)** |
| --- | --- | --- | --- | --- |
| ***Eulemur*** |  | **2** | **12** | **14** |
| *Eulemur rubriventer* | Red-bellied Lemur | 0 | 12 | 12 |
| *Eulemur rufus* | Red Lemur | 2 | 0 | 2 |
| ***Lemur*** |  | **3** | **10** | **13** |
| *Lemur catta* | Ring-tailed Lemur | 3 | 10 | 13 |
| ***Gorilla*** |  | **8** | **11** | **19** |
| *Gorilla gorilla* | Western Gorilla | 8 | 11 | 19 |
| ***Ateles*** |  | **2** | **8** | **10** |
| *Ateles belzebuth* | White-bellied Spider Monkey | 0 | 5 | 5 |
| *Ateles fusciceps* | Black-headed Spider Monkey | 2 | 0 | 2 |
| *Ateles hybridus* | Brown Spider Monkey | 0 | 3 | 3 |
| ***Antidorcas*** |  | **5** | **4** | **9** |
| *Antidorcas marsupialis* | Springbok | 5 | 4 | 9 |
| ***Colobus*** |  | **3** | **8** | **11** |
| *Colubus angolensis* | Black and White Colobus | 2 | 0 | 2 |
| *Colubus guereza* | Mantled Guereza | 1 | 8 | 9 |
| ***Cercopithecus*** |  | **5** | **8** | **13** |
| *Cercopithecus ascanius* | Red-tailed Monkey | 1 | 8 | 9 |
| *Cercopithecus cephus* | Moustached Guenon | 2 | 0 | 2 |
| *Cercopithecus neglectus* | De Brazza’s Monkey | 1 | 0 | 1 |
| *Cercopithecus wolfi* | Wolf’s Guenon | 1 | 0 | 1 |
| ***Connochaetes*** |  | **6** | **2** | **8** |
| *Connochaetes gnou* | Black Wildebeest | 1 | 0 | 1 |
| *Connochaetes taurinus* | Blue Wildebeest | 5 | 2 | 7 |
| ***Orycteropus*** |  | **18** | **5** | **23** |
| *Orycteropus afer* | Aardvark | 18 | 5 | 23 |
| ***Ceratotherium_Diceros*** |  | **9** | **4** | **13** |
| *Ceratotherium simum* | White Rhinoceros | 3 | 3 | 6 |
| *Diceros bicornis* | Black Rhinoceros | 6 | 1 | 7 |
| ***Myrmecophaga*** |  | **11** | **30** | **41** |
| *Myrmecophaga tridactyla* | Giant Anteater | 11 | 30 | 41 |
| ***Equus*** |  | **22** | **9** | **31** |
| *Equus asinus* | African Wild Ass | 5 | 0 | 5 |
| *Equus quagga* | Plains Zebra | 4 | 2 | 6 |
| *Equus grevyi* | Greyvi’s Zebra | 3 | 0 | 3 |
| *Equus hemionus* | Onager | 3 | 0 | 3 |
| *Equus przewalskii* | Przewalski’s Horse | 4 | 4 | 8 |
| *Equus zebra* | Mountain Zebra | 3 | 3 | 6 |
| ***Aepyceros*** |  | **3** | **3** | **6** |
| *Aepyceros melampus* | Impala | 3 | 3 | 6 |
| ***Giraffa*** |  | **4** | **2** | **6** |
| *Giraffa camelopardalis* | Giraffe | 4 | 2 | 6 |
| ***Hippotragus*** |  | **5** | **2** | **7** |
| *Hippotragus equinus* | Roan Antelope | 1 | 0 | 1 |
| *Hippotragus niger* | Sable Antelope | 4 | 2 | 6 |
| **Diet Type** |  |  |  |  |
| **Carnivore** |  | **35** | **41** | **76** |
| Canis lupus | Wolf | 4 | 0 | 4 |
| Lycaon pictus | African Wild Dog | 1 | 4 | 5 |
| Acinonyx jubatus | Cheetah | 1 | 2 | 3 |
| Myrmecophaga tridactyla | Giant Anteater | 11 | 30 | 41 |
| Orycteropus afer | Aardvark | 18 | 5 | 23 |
| **Herbivore** |  | **67** | **59** | **126** |
| Aepyceros melampus | Impala | 3 | 3 | 6 |
| Antidorcas marsupialis | Springbok | 5 | 4 | 9 |
| Connochaetes gnou | Black Wildebeest | 1 | 0 | 1 |
| Connochaetes taurinus | Blue Wildebeest | 5 | 2 | 7 |
| Hippotragus equinus | Roan Antelope | 1 | 0 | 1 |
| Hippotragus niger | Sable Antelope | 4 | 2 | 6 |
| Giraffa camelopardalis | Giraffe | 4 | 2 | 6 |
| Equus asinus | African Wild Ass | 5 | 0 | 5 |
| Equus quagga | Plains Zebra | 4 | 2 | 6 |
| Equus grevyi | Greyvi’s Zebra | 3 | 0 | 3 |
| Equus hemionus | Onager | 3 | 0 | 3 |
| Equus przewalskii | Przewalski’s Horse | 4 | 4 | 8 |
| Equus zebra | Mountain Zebra | 3 | 3 | 6 |
| Ceratotherium simum | White Rhinoceros | 3 | 3 | 6 |
| Diceros bicornis | Black Rhinoceros | 6 | 1 | 7 |
| Gorilla gorilla | Western Gorilla | 8 | 11 | 19 |
| Eulemur rubriventer | Red-bellied Lemur | 0 | 12 | 12 |
| Eulemur rufus | Red Lemur | 2 | 0 | 2 |
| Lemur catta | Ring-tailed Lemur | 3 | 10 | 13 |
| **Omnivore** |  | **13** | **92** | **105** |
| Phacochoerus africanus | Common Warthog | 1 | 4 | 5 |
| Alouatta caraya | Black Howler | 0 | 12 | 12 |
| Alouatta palliata | Mantled Howler | 0 | 12 | 12 |
| Alouatta pigra | Guatemalan Black Howler | 2 | 13 | 15 |
| Alouatta seniculus | Venezuelan Red Howler | 0 | 10 | 10 |
| Ateles belzebuth | White-bellied Spider Monkey | 0 | 5 | 5 |
| Ateles fusciceps | Black-headed Spider Monkey | 2 | 0 | 2 |
| Ateles hybridus | Brown Spider Monkey | 0 | 3 | 3 |
| Cercopithecus ascanius | Red-tailed Monkey | 1 | 8 | 9 |
| Cercopithecus cephus | Moustached Guenon | 2 | 0 | 2 |
| Cercopithecus neglectus | De Brazza’s Monkey | 1 | 0 | 1 |
| Cercopithecus wolfi | Wolf’s Guenon | 1 | 0 | 1 |
| Colobus angolensis | Black and White Colobus | 2 | 0 | 2 |
| Colobus guereza | Mantled Guereza | 1 | 8 | 9 |
| Papio anubis | Olive Baboon | 0 | 7 | 7 |
| Papio hamadryas | Hamadryas Baboon | 0 | 8 | 8 |
| Papio ursinus | Chacma Baboon | 0 | 2 | 2 |
| **Fermenter Type** |  |  |  |  |
| **Hindgut** |  | **42** | **75** | **117** |
| Equus asinus | African Wild Ass | 5 | 0 | 5 |
| Equus quagga | Plains Zebra | 4 | 2 | 6 |
| Equus grevyi | Greyvi’s Zebra | 3 | 0 | 3 |
| Equus hemionus | Onager | 3 | 0 | 3 |
| Equus przewalskii | Przewalski’s Horse | 4 | 4 | 8 |
| Equus zebra | Mountain Zebra | 3 | 3 | 6 |
| Ceratotherium simum | White Rhinoceros | 3 | 3 | 6 |
| Diceros bicornis | Black Rhinoceros | 6 | 1 | 7 |
| Gorilla gorilla | Western Gorilla | 8 | 11 | 19 |
| Phacochoerus africanus | Common Warthog | 1 | 4 | 5 |
| Alouatta caraya | Black Howler | 0 | 12 | 12 |
| Alouatta palliata | Mantled Howler | 0 | 12 | 12 |
| Alouatta pigra | Guatemalan Black Howler | 2 | 13 | 15 |
| Alouatta seniculus | Venezuelan Red Howler | 0 | 10 | 10 |
| **Foregut** |  | **26** | **21** | **47** |
| Aepyceros melampus | Impala | 3 | 3 | 6 |
| Antidorcas marsupialis | Springbok | 5 | 4 | 9 |
| Connochaetes gnou | Black Wildebeest | 1 | 0 | 1 |
| Connochaetes taurinus | Blue Wildebeest | 5 | 2 | 7 |
| Hippotragus equinus | Roan Antelope | 1 | 0 | 1 |
| Hippotragus niger | Sable Antelope | 4 | 2 | 6 |
| Giraffa camelopardalis | Giraffe | 4 | 2 | 6 |
| Colobus angolensis | Black and White Colobus | 2 | 0 | 2 |
| Colobus guereza | Mantled Guereza | 1 | 8 | 9 |
| **Neither** |  | **47** | **96** | **143** |
| Canis lupus | Wolf | 4 | 0 | 4 |
| Lycaon pictus | African Wild Dog | 1 | 4 | 5 |
| Acinonyx jubatus | Cheetah | 1 | 2 | 3 |
| Myrmecophaga tridactyla | Giant Anteater | 11 | 30 | 41 |
| Orycteropus afer | Aardvark | 18 | 5 | 23 |
| Eulemur rubriventer | Red-bellied Lemur | 0 | 12 | 12 |
| Eulemur rufus | Red Lemur | 2 | 0 | 2 |
| Lemur catta | Ring-tailed Lemur | 3 | 10 | 13 |
| Ateles belzebuth | White-bellied Spider Monkey | 0 | 5 | 5 |
| Ateles fusciceps | Black-headed Spider Monkey | 2 | 0 | 2 |
| Ateles hybridus | Brown Spider Monkey | 0 | 3 | 3 |
| Cercopithecus ascanius | Red-tailed Monkey | 1 | 8 | 9 |
| Cercopithecus cephus | Moustached Guenon | 2 | 0 | 2 |
| Cercopithecus neglectus | De Brazza’s Monkey | 1 | 0 | 1 |
| Cercopithecus wolfi | Wolf’s Guenon | 1 | 0 | 1 |
| Papio anubis | Olive Baboon | 0 | 7 | 7 |
| Papio hamadryas | Hamadryas Baboon | 0 | 8 | 8 |
| Papio ursinus | Chacma Baboon | 0 | 2 | 2 |
