# Supplementary material for: The Effects of Captivity on the Mammalian Gut Microbiome
Source: Integr Comp Biol. 2017 Aug 7;57(4):690–704. doi: 10.1093/icb/icx090 (PMC5978021; doi:10.1093/icb/icx090)
Supplement: Supplementary Data [file icx090_supp.zip › icx090_SuppTable_4.docx]

Supplementary Table S4. Results of 2-way Adonis anlayes to examine the effects of collection site and captive/wild for 5 mammal taxa that were collected from multiple captive sites: *Antidorcas marsupialis* (springbok), *Ceratotherium/Diceros* (black and white rhinoceros), *Gorilla gorilla*, *Myrmecophaga tridactyla* (anteater), and *Orycteropus afer* (aardvark). Results indicated that for all 5 taxa, both collection site and captive/wild were significant factors explaining variation in bacterial gut beta-diversity (see Figure S1). In all cases, the captive/wild factor had a stronger effect size (mean sum of squares) relative to collection site; in the case of gorillas, the effect size for captive/wild was 4.3 times greater than collection site.

| *Antidorcas marsupialis* |  | **Df** | **Sums Of Sqs** | **MeanSqs** | **F.Model** | **R2** | **Pr(>F)** |
| --- | --- | --- | --- | --- | --- | --- | --- |
|  | Captive_Wild | 1 | 0.80958 | 0.80958 | 3.7835 | 0.31239 | 0.001 |
|  | Collection Site | 2 | 0.71206 | 0.35603 | 1.6638 | 0.27476 | 0.043 |
|  | Residual | 5 | 1.06989 | 0.21398 |  | 0.41284 |  |
|  | Total | 8 | 2.59153 |  |  |  |  |
| *Ceratotherium/Diceros* |  |  |  |  |  |  |  |
|  | Captive_Wild | 1 | 0.7977 | 0.79767 | 3.4951 | 0.16327 | 0.001 |
|  | Collection Site | 3 | 2.2621 | 0.75403 | 3.3039 | 0.46302 | 0.001 |
|  | Residual | 8 | 1.8258 | 0.22823 |  | 0.37371 |  |
|  | Total | 12 | 4.8856 |  |  |  |  |
| *Gorilla gorilla* |  |  |  |  |  |  |  |
|  | Captive_Wild | 1 | 1.9603 | 1.96028 | 8.2021 | 0.30318 | 0.001 |
|  | Collection Site | 2 | 0.9204 | 0.46021 | 1.9256 | 0.14236 | 0.013 |
|  | Residual | 15 | 3.585 | 0.239 |  | 0.55446 |  |
|  | Total | 18 | 6.4657 |  |  |  |  |
| *Myrmecophaga tridactyla* |  |  |  |  |  |  |  |
|  | Captive_Wild | 1 | 1.4845 | 1.48448 | 3.874 | 0.08599 | 0.001 |
|  | Collection Site | 2 | 1.6009 | 0.80043 | 2.0888 | 0.09273 | 0.001 |
|  | Residual | 37 | 14.1781 | 0.38319 |  | 0.82128 |  |
|  | Total | 40 | 17.2635 |  |  |  |  |
| *Orycteropus afer* |  |  |  |  |  |  |  |
|  | Captive_Wild | 1 | 1.6759 | 1.676 | 4.889 | 0.1737 | 0.001 |
|  | Collection Site | 1 | 1.1168 | 1.1168 | 3.2578 | 0.11574 | 0.001 |
|  | Residual | 20 | 6.8561 | 0.3428 |  | 0.71056 |  |
|  | Total | 22 | 9.6488 |  |  |  |  |
