# Supplementary material for: The Effects of Captivity on the Mammalian Gut Microbiome
Source: Integr Comp Biol. 2017 Aug 7;57(4):690–704. doi: 10.1093/icb/icx090 (PMC5978021; doi:10.1093/icb/icx090)
Supplement: Supplementary Data [file icx090_supp.zip › icx090_SuppTable_5.docx]

Supplementary Table S6. Summary statistics for bacterial taxa that shift in abundance from captive to wild across all mammals (FDR p-value ≥ 0.05, Mann-Whitney U tests). These statistics are visualized in Figure 4.

| **Taxonomic level** | **FDR pvals (Mann-Whitney *U* test)** | **Wild mean relative abundance (RA)** | **Captive mean RA** | **Phlya** | **Class** | **Family** | **Genus** | **Species** |
| --- | --- | --- | --- | --- | --- | --- | --- | --- |
| Phylum Actinobacteria | 0.0032 | 0.0410 | 0.0178 | Actinobacteria | -- | -- | -- | -- |
| Class Coriobacteriia | 0.0015 | 0.0141 | 0.0048 | Actinobacteria | Coriobacteriia | -- | -- | -- |
| Phylum Bacteroidetes | 0.0030 | 0.2495 | 0.3005 | Bacteroidetes | -- | -- | -- | -- |
| Class Bacteroidia | 0.0165 | 0.2203 | 0.2583 | Bacteroidetes | Bacteroidia | -- | -- | -- |
| Genus CF231 | 0.0000 | 0.0024 | 0.0104 | Bacteroidetes | Bacteroidia | [Paraprevotellaceae] | CF231 | -- |
| Genus Prevotella | 0.0215 | 0.0731 | 0.0458 | Bacteroidetes | Bacteroidia | Prevotellaceae | Prevotella | -- |
| Class Flavobacteriia | 0.0017 | 0.0087 | 0.0151 | Bacteroidetes | Flavobacteriia | -- | -- | -- |
| Phylum Cyanobacteria | 0.0000 | 0.0187 | 0.0049 | Cyanobacteria | -- | -- | -- | -- |
| Class 4C0d-2 | 0.0000 | 0.0183 | 0.0048 | Cyanobacteria | 4C0d-2 | -- | -- | -- |
| Class Bacilli | 0.0000 | 0.0478 | 0.0845 | Firmicutes | Bacilli | -- | -- | -- |
| Genus Streptococcus | 0.0000 | 0.0009 | 0.0277 | Firmicutes | Bacilli | Streptococcaceae | Streptococcus | -- |
| OTU sp_luteciae | 0.0000 | 0.0008 | 0.0230 | Firmicutes | Bacilli | Streptococcaceae | Streptococcus | luteciae |
| Class Clostridia | 0.0083 | 0.3289 | 0.2769 | Firmicutes | Clostridia | -- | -- | -- |
| Genus Clostridium | 0.0042 | 0.0127 | 0.0281 | Firmicutes | Clostridia | Clostridiaceae | Clostridium | -- |
| Genus Coprococcus | 0.0000 | 0.0133 | 0.0072 | Firmicutes | Clostridia | Lachnospiraceae | Coprococcus | -- |
| Genus Faecalibacterium | 0.0000 | 0.0174 | 0.0076 | Firmicutes | Clostridia | Ruminococcaceae | Faecalibacterium | -- |
| Genus Oscillospira | 0.0157 | 0.0181 | 0.0118 | Firmicutes | Clostridia | Ruminococcaceae | Oscillospira | -- |
| Class Erysipelotrichi | 0.0049 | 0.0370 | 0.0276 | Firmicutes | Erysipelotrichi | -- | -- | -- |
| Class Alphaproteobacteria | 0.0001 | 0.0224 | 0.0076 | Proteobacteria | Alphaproteobacteria | -- | -- | -- |
| Class Betaproteobacteria | 0.0002 | 0.0162 | 0.0090 | Proteobacteria | Betaproteobacteria | -- | -- | -- |
| Class Gammaproteobacteria | 0.0014 | 0.0384 | 0.0929 | Proteobacteria | Gammaproteobacteria | -- | -- | -- |
| Class Acinetobacter | 0.0252 | 0.0109 | 0.0181 | Proteobacteria | Gammaproteobacteria | Moraxellaceae | Acinetobacter | -- |
| OTU (Enterobacteriaceae) | 0.0027 | 0.0107 | 0.0418 | Proteobacteria | Gammaproteobacteria | Enterobacteriaceae | | s__ |
| Phylum Spirochaetes | 0.0001 | 0.0127 | 0.0163 | Spirochaetes | -- | -- | -- | -- |
| Class Spirochaetes | 0.0001 | 0.0126 | 0.0153 | Spirochaetes | Spirochaetes | -- | -- | -- |
| Class Treponema | 0.0000 | 0.0098 | 0.0136 | Spirochaetes | Spirochaetes | Spirochaetaceae | Treponema | -- |
| Phylum Tenericutes | 0.0035 | 0.0358 | 0.0211 | Tenericutes | -- | -- | -- | -- |
| Class Mollicutes | 0.0036 | 0.0337 | 0.0195 | Tenericutes | Mollicutes | -- | -- | -- |
| Class Verruco-5 | 0.0229 | 0.0123 | 0.0225 | Verrucomicrobia | Verruco-5 | -- | -- | -- |

Supplementary Table S5. Summary statistics for  bacterial taxa that shift in abundance from captive to wild  within each mammal genus (FDR p-value ≥ 0.05, Mann-Whitney U tests). These statistics are visualized in Figure 5.

| **Host genus** | **FDR pvals (Mann-Whitney *U* test)** | **Wild mean relative abundance (RA)** | **Captive mean RA** | **Phlya** | **Class** | **Family** | **Genus** | **Species** |
| --- | --- | --- | --- | --- | --- | --- | --- | --- |
| Ceratotherium_Diceros | 0.0282 | 0.0226 | 0.0000 | Bacteroidetes | Bacteroidia | [Paraprevotellaceae] | -- | -- |
| Cercopithecus | 0.0274 | 0.0210 | 0.0000 | Actinobacteria | Coriobacteriia | Coriobacteriaceae | -- | -- |
| Cercopithecus | 0.0375 | 0.0000 | 0.0113 | Bacteroidetes | Bacteroidia | S24-7 | -- | -- |
| Cercopithecus | 0.0274 | 0.0175 | 0.0000 | Bacteroidetes | Bacteroidia | Prevotellaceae | Prevotella | -- |
| Cercopithecus | 0.0274 | 0.0186 | 0.0000 | Bacteroidetes | Bacteroidia | Prevotellaceae | Prevotella | copri |
| Cercopithecus | 0.0294 | 0.0007 | 0.0102 | Firmicutes | Clostridia | Christensenellaceae | -- | -- |
| Cercopithecus | 0.0256 | 0.0112 | 0.0020 | Firmicutes | Clostridia | Ruminococcaceae | Oscillospira | -- |
| Cercopithecus | 0.0256 | 0.0235 | 0.0012 | Firmicutes | Erysipelotrichi | Erysipelotrichaceae | -- | -- |
| Colobus | 0.0396 | 0.0000 | 0.0548 | Bacteroidetes | Bacteroidia | [Paraprevotellaceae] | CF231 | -- |
| Colobus | 0.0396 | 0.0000 | 0.0101 | Bacteroidetes | Bacteroidia | -- | -- | -- |
| Colobus | 0.0408 | 0.0000 | 0.0346 | Proteobacteria | -- | -- | -- | -- |
| Colobus | 0.0408 | 0.0004 | 0.0115 | Verrucomicrobia | Verruco-5 | RFP12 | -- | -- |
| Equus | 0.0139 | 0.0122 | 0.0000 | Bacteroidetes | Bacteroidia | Porphyromonadaceae | Paludibacter | -- |
| Eulemur | 0.0028 | 0.0000 | 0.1066 | Bacteroidetes | Bacteroidia | -- | -- | -- |
| Eulemur | 0.0028 | 0.0000 | 0.0616 | Bacteroidetes | Bacteroidia | Prevotellaceae | Prevotella | copri |
| Eulemur | 0.0028 | 0.0000 | 0.0429 | Bacteroidetes | Bacteroidia | Prevotellaceae | Prevotella | -- |
| Eulemur | 0.0028 | 0.0000 | 0.0298 | Bacteroidetes | Bacteroidia | Bacteroidaceae | Bacteroides | -- |
| Eulemur | 0.0028 | 0.0000 | 0.0270 | Bacteroidetes | Bacteroidia | -- | -- | -- |
| Eulemur | 0.0028 | 0.0000 | 0.0250 | Bacteroidetes | Bacteroidia | Rikenellaceae | -- | -- |
| Eulemur | 0.0084 | 0.0000 | 0.0200 | Bacteroidetes | Bacteroidia | -- | -- | -- |
| Eulemur | 0.0028 | 0.0000 | 0.0103 | Bacteroidetes | Bacteroidia | [Paraprevotellaceae] | YRC22 | -- |
| Eulemur | 0.0028 | 0.0000 | 0.0101 | Bacteroidetes | Bacteroidia | Bacteroidaceae | Bacteroides | uniformis |
| Eulemur | 0.0028 | 0.0000 | 0.0609 | Firmicutes | Clostridia | Ruminococcaceae | -- | -- |
| Eulemur | 0.0028 | 0.0000 | 0.0369 | Firmicutes | Clostridia | Lachnospiraceae | Blautia | -- |
| Eulemur | 0.0028 | 0.0000 | 0.0187 | Firmicutes | Clostridia | Ruminococcaceae | Ruminococcus | bromii |
| Eulemur | 0.0028 | 0.0000 | 0.0179 | Firmicutes | Clostridia | -- | -- | -- |
| Eulemur | 0.0028 | 0.0000 | 0.0162 | Firmicutes | Clostridia | Lachnospiraceae | -- | -- |
| Eulemur | 0.0028 | 0.0000 | 0.0145 | Firmicutes | Clostridia | Lachnospiraceae | -- | -- |
| Eulemur | 0.0028 | 0.0000 | 0.0124 | Firmicutes | Erysipelotrichi | Erysipelotrichaceae | -- | -- |
| Eulemur | 0.0483 | 0.0000 | 0.0110 | Firmicutes | Clostridia | Ruminococcaceae | Ruminococcus | -- |
| Eulemur | 0.0474 | 0.0198 | 0.0023 | Firmicutes | Clostridia | Veillonellaceae | Phascolarctobacterium | -- |
| Eulemur | 0.0474 | 0.0248 | 0.0001 | Proteobacteria | Betaproteobacteria | Alcaligenaceae | Sutterella | -- |
| Eulemur | 0.0474 | 0.0161 | 0.0005 | Verrucomicrobia | Verruco-5 | -- | -- | -- |
| Gorilla | 0.0009 | 0.0117 | 0.0000 | Actinobacteria | Coriobacteriia | Coriobacteriaceae | -- | -- |
| Gorilla | 0.0178 | 0.0170 | 0.0015 | Actinobacteria | Coriobacteriia | Coriobacteriaceae | -- | -- |
| Gorilla | 0.0015 | 0.0253 | 0.0000 | Actinobacteria | Actinobacteria | Bifidobacteriaceae | Bifidobacterium | adolescentis |
| Gorilla | 0.0478 | 0.0000 | 0.0230 | Bacteroidetes | Bacteroidia | BS11 | -- | -- |
| Gorilla | 0.0009 | 0.0122 | 0.0000 | Bacteroidetes | Bacteroidia | Prevotellaceae | Prevotella | -- |
| Gorilla | 0.0026 | 0.0137 | 0.0000 | Bacteroidetes | Bacteroidia | -- | -- | -- |
| Gorilla | 0.0009 | 0.0181 | 0.0000 | Bacteroidetes | Bacteroidia | Prevotellaceae | Prevotella | -- |
| Gorilla | 0.0251 | 0.0232 | 0.0000 | Bacteroidetes | Bacteroidia | -- | -- | -- |
| Gorilla | 0.0151 | 0.0455 | 0.0075 | Bacteroidetes | -- | -- | -- | -- |
| Gorilla | 0.0257 | 0.0027 | 0.0299 | Firmicutes | Clostridia | Clostridiaceae | Sarcina | -- |
| Gorilla | 0.0060 | 0.0006 | 0.0277 | Firmicutes | Bacilli | Lactobacillaceae | Lactobacillus | -- |
| Gorilla | 0.0070 | 0.0000 | 0.0246 | Firmicutes | Bacilli | Lactobacillaceae | Lactobacillus | -- |
| Gorilla | 0.0028 | 0.0000 | 0.0174 | Firmicutes | Bacilli | Lactobacillaceae | Lactobacillus | -- |
| Gorilla | 0.0026 | 0.0122 | 0.0000 | Firmicutes | Clostridia | Ruminococcaceae | -- | -- |
| Gorilla | 0.0009 | 0.0126 | 0.0000 | Firmicutes | Clostridia | Lachnospiraceae | -- | -- |
| Gorilla | 0.0009 | 0.0127 | 0.0000 | Firmicutes | Clostridia | Lachnospiraceae | Pseudobutyrivibrio | -- |
| Gorilla | 0.0009 | 0.0138 | 0.0000 | Firmicutes | Clostridia | [Mogibacteriaceae] | Mogibacterium | -- |
| Gorilla | 0.0033 | 0.0177 | 0.0000 | Firmicutes | Clostridia | Lachnospiraceae | Coprococcus | -- |
| Gorilla | 0.0009 | 0.0440 | 0.0000 | Firmicutes | Clostridia | Ruminococcaceae | -- | -- |
| Gorilla | 0.0478 | 0.0000 | 0.0147 | Proteobacteria | Gammaproteobacteria | Enterobacteriaceae | -- | -- |
| Gorilla | 0.0008 | 0.0205 | 0.0002 | Spirochaetes | Spirochaetes | Spirochaetaceae | Treponema | -- |
| Gorilla | 0.0009 | 0.0102 | 0.0000 | Verrucomicrobia | Verruco-5 | RFP12 | -- | -- |
| Gorilla | 0.0066 | 0.0116 | 0.0000 | Verrucomicrobia | Verruco-5 | -- | -- | -- |
| Lemur | 0.0196 | 0.0000 | 0.0192 | Bacteroidetes | Bacteroidia | [Paraprevotellaceae] | -- | -- |
| Lemur | 0.0303 | 0.0105 | 0.0000 | Bacteroidetes | Bacteroidia | -- | -- | -- |
| Lemur | 0.0303 | 0.0114 | 0.0000 | Bacteroidetes | Bacteroidia | -- | -- | -- |
| Lemur | 0.0303 | 0.0196 | 0.0000 | Bacteroidetes | Bacteroidia | Bacteroidaceae | -- | -- |
| Lemur | 0.0303 | 0.0196 | 0.0000 | Bacteroidetes | Bacteroidia | Prevotellaceae | Prevotella | -- |
| Lemur | 0.0303 | 0.0202 | 0.0000 | Bacteroidetes | Bacteroidia | Prevotellaceae | Prevotella | -- |
| Lemur | 0.0303 | 0.0212 | 0.0000 | Bacteroidetes | Bacteroidia | -- | -- | -- |
| Lemur | 0.0303 | 0.0279 | 0.0000 | Bacteroidetes | Bacteroidia | Prevotellaceae | Prevotella | melaninogenica |
| Lemur | 0.0303 | 0.0101 | 0.0000 | Firmicutes | Clostridia | Lachnospiraceae | -- | -- |
| Lemur | 0.0303 | 0.0137 | 0.0000 | Firmicutes | Clostridia | Ruminococcaceae | -- | -- |
| Lemur | 0.0303 | 0.0137 | 0.0000 | Firmicutes | Clostridia | Lachnospiraceae | Shuttleworthia | -- |
| Lemur | 0.0303 | 0.0151 | 0.0000 | Firmicutes | Clostridia | Lachnospiraceae | -- | -- |
| Lemur | 0.0303 | 0.0190 | 0.0000 | Firmicutes | Erysipelotrichi | Erysipelotrichaceae | RFN20 | -- |
| Lemur | 0.0303 | 0.0213 | 0.0000 | Firmicutes | Erysipelotrichi | Erysipelotrichaceae | RFN20 | -- |
| Lemur | 0.0303 | 0.0367 | 0.0000 | Firmicutes | Clostridia | Lachnospiraceae | -- | -- |
| Lemur | 0.0303 | 0.0695 | 0.0000 | Firmicutes | Erysipelotrichi | Erysipelotrichaceae | RFN20 | -- |
| Myrmecophaga | 0.0084 | 0.0131 | 0.0000 | Actinobacteria | Actinobacteria | -- | -- | -- |
| Myrmecophaga | 0.0025 | 0.0331 | 0.0000 | Actinobacteria | Actinobacteria | Thermomonosporaceae | Actinomadura | vinacea |
| Myrmecophaga | 0.0464 | 0.0151 | 0.0000 | Bacteroidetes | Flavobacteriia | Flavobacteriaceae | -- | -- |
| Myrmecophaga | 0.0338 | 0.0000 | 0.0438 | Firmicutes | Bacilli | Streptococcaceae | Streptococcus | luteciae |
| Myrmecophaga | 0.0000 | 0.0000 | 0.0234 | Firmicutes | Erysipelotrichi | Erysipelotrichaceae | [Eubacterium] | biforme |
| Myrmecophaga | 0.0005 | 0.0000 | 0.0175 | Firmicutes | Clostridia | Veillonellaceae | Megasphaera | -- |
| Myrmecophaga | 0.0023 | 0.0000 | 0.0171 | Firmicutes | Clostridia | Clostridiaceae | Clostridium | hiranonis |
| Myrmecophaga | 0.0000 | 0.0000 | 0.0153 | Firmicutes | Erysipelotrichi | Erysipelotrichaceae | Bulleidia | -- |
| Myrmecophaga | 0.0001 | 0.0002 | 0.0105 | Firmicutes | Bacilli | Turicibacteraceae | Turicibacter | -- |
| Myrmecophaga | 0.0128 | 0.0103 | 0.0034 | Firmicutes | Bacilli | Streptococcaceae | Lactococcus | garvieae |
| Myrmecophaga | 0.0009 | 0.1384 | 0.0003 | Firmicutes | Bacilli | Planococcaceae | Rummeliibacillus | -- |
| Myrmecophaga | 0.0343 | 0.0176 | 0.0000 | Proteobacteria | Alphaproteobacteria | Brucellaceae | Ochrobactrum | gallinifaecis |
| Myrmecophaga | 0.0203 | 0.0202 | 0.0000 | Proteobacteria | Betaproteobacteria | Alcaligenaceae | Alcaligenes | faecalis |
| Myrmecophaga | 0.0342 | 0.0203 | 0.0001 | Proteobacteria | Betaproteobacteria | Comamonadaceae | Comamonas | -- |
| Orycteropus | 0.0025 | 0.0206 | 0.0017 | Actinobacteria | Coriobacteriia | Coriobacteriaceae | Collinsella | stercoris |
| Orycteropus | 0.0018 | 0.0201 | 0.0004 | Bacteroidetes | Bacteroidia | Bacteroidaceae | Bacteroides | eggerthii |
| Orycteropus | 0.0025 | 0.0352 | 0.0009 | Bacteroidetes | Bacteroidia | Bacteroidaceae | Bacteroides | -- |
| Orycteropus | 0.0241 | 0.0353 | 0.0004 | Bacteroidetes | Bacteroidia | [Paraprevotellaceae] | [Prevotella] | -- |
| Orycteropus | 0.0023 | 0.0545 | 0.0002 | Bacteroidetes | Bacteroidia | Porphyromonadaceae | Parabacteroides | distasonis |
| Orycteropus | 0.0396 | 0.0000 | 0.0198 | Firmicutes | Erysipelotrichi | Erysipelotrichaceae | [Eubacterium] | biforme |
| Orycteropus | 0.0025 | 0.0100 | 0.0000 | Firmicutes | Clostridia | Lachnospiraceae | Blautia | -- |
| Orycteropus | 0.0025 | 0.0125 | 0.0000 | Firmicutes | Clostridia | Lachnospiraceae | Blautia | -- |
| Orycteropus | 0.0018 | 0.0136 | 0.0002 | Firmicutes | Clostridia | Ruminococcaceae | -- | -- |
| Orycteropus | 0.0025 | 0.0153 | 0.0000 | Firmicutes | Erysipelotrichi | Erysipelotrichaceae | Clostridium | -- |
| Orycteropus | 0.0020 | 0.0242 | 0.0004 | Firmicutes | Clostridia | -- | -- | -- |
| Orycteropus | 0.0001 | 0.0371 | 0.0000 | Firmicutes | Clostridia | Lachnospiraceae | -- | -- |
| Orycteropus | 0.0185 | 0.0428 | 0.0000 | Firmicutes | Clostridia | -- | -- | -- |
| Orycteropus | 0.0185 | 0.0486 | 0.0000 | Firmicutes | Erysipelotrichi | Erysipelotrichaceae | Allobaculum | -- |
| Orycteropus | 0.0001 | 0.0935 | 0.0000 | Firmicutes | Clostridia | Lachnospiraceae | Blautia | -- |
| Orycteropus | 0.0107 | 0.0932 | 0.0000 | Fusobacteria | Fusobacteriia | Fusobacteriaceae | -- | -- |
| Orycteropus | 0.0378 | 0.0000 | 0.0179 | Proteobacteria | Alphaproteobacteria | Phyllobacteriaceae | -- | -- |
| Orycteropus | 0.0006 | 0.0151 | 0.0000 | Proteobacteria | Betaproteobacteria | -- | -- | -- |
| Orycteropus | 0.0023 | 0.0191 | 0.0006 | Proteobacteria | Betaproteobacteria | -- | -- | -- |
